# Cheap and sensitive polymer/bismuth film modified electrode for simultaneous determination of Pb(II) and Cd(II) ions

**DOI:** 10.1016/j.heliyon.2021.e08215

**Published:** 2021-10-19

**Authors:** Alemayehu Yifru, Gossa Dare, Taye B. Demissie, Solomon Mehretie, Shimelis Admassie

**Affiliations:** a Department of Chemistry, Addis Ababa University, PO Box 1176, Addis Ababa, Ethiopia; b Materials Science Program, Department of Chemistry, Addis Ababa University, PO Box 1176, Addis Ababa, Ethiopia

**Keywords:** 8-Aminonaphthalene-2-sulphonic acid, Square wave anodic stripping voltammetry, Pb(II), Cd(II)

## Abstract

Different aminonaphetalenesulphonic acid derivatives like 5-aminonaphthalene-1-sulphonic acid (5AN1SA), 2-aminonaphthalene-1-sulphonic acid (2AN1SA), 8-aminonaphthalene-2-sulphonic acid (8AN2SA) and 4-amino-3-hydroxynaphthalene-1-sulphonic acid (4A3HN1SA) were used to construct polymer/bismuth film modified electrode for simultaneous determination of Pb(II) and Cd(II) ions with the aim of developing a cheaper and sensitive electrode that could possibly replace Nafion. Among the different modified electrodes, poly (8AN2SA)/bismuth film modified electrodes showed the highest electrochemical response for both ions. These electrochemical results were also supported by density functional theory (DFT) calculations. Based on these experimental and theoretical results, poly (8AN2SA)/bismuth film glassy carbon modified electrode was further investigated to develop a simple and sensitive electrochemical method for the simultaneous determination of Pb(II) and Cd(II) ions. After optimizing the different experimental parameters, the proposed method gave a linear range of 1–40 μg/L with the detection limit of 0.38 and 0.08 μg/L for the simultaneous determination of Pb(II) and Cd(II) ions, respectively.

## 1 Introduction

Environmental pollution as a result of population explosion and rapid industrialization is becoming a setback for a sustainable development particularly in developing countries [1, 2]. Among the various pollutants, heavy metals such as arsenic, lead, cadmium, chromium, and mercury, which are non-degradable and persistent in the environment, are of great concern to humans [3].

Major sources of pollution of the environment by lead are anthropogenic including emissions of automobiles; lead paints, pipes and batteries; lead-glazed ceramics and containers; fertilizers, pesticides, cosmetics and toys [4]. Lead poisoning causes severe dysfunction of different organs like the kidneys, liver, reproductive system, and mental retardation, especially in children. Lead is listed as the second top toxic pollutant in the priority next to arsenic by the Agency for Toxic Substances and Disease Registry (ATSDR) [5]. The Institute for Health Metrics and Evaluation has estimated that in 2017 lead exposure accounted for 1.06 million deaths and 24.4 million years lost to disability and death due to long-term health effects, with the highest burden in developing regions [6]. Hence, lead is considered by the World Health Organization (WHO) as one of the ten chemicals of major public health concern requiring immediate actions and set threshold limits of 10 ppb for lead in drinking water [6]. Despite regulatory, remediation and public awareness campaigns, lead contamination continued to be a serious concern even for the developed nations as documented in recent Flint, Michigan and Washington DC water crises [7, 8].

Cadmium which is released mainly from batteries, coatings and platings, stabilizers for plastics, fossil fuel combustion, phosphate fertilizer, and waste incinerationis classified as a Group 1 human carcinogen [9, 10]. Cadmium exposure causes disruption of presynaptic function, olfactory dysfunction, nasal epithelial damage, behavioral change, tubular proteinuria, Alzheimer's disease, Parkinson's disease and lung cancer [11]. The cause of Itai-Itai disease in Japan, which softens the bones as a result of renal tubular dysfunction, is also known to be chronic cadmium poisoning [12]. Besides, large and uncontrolled dumping of lead and cadmium-containing wastes from used batteries and other metal-containing e-wastes in developing countries are threatening the lives of thousands of people [13, 14, 15]. The WHO threshold limits for cadmium in drinking water is 3 ppb further lower than that of lead [6].

Hence, a cheap, sensitive, selective, and environmentally friendly analytical methods and materials affordable for developing countries are of utmost importance to regularly monitor the amounts of these toxic metals in different matrices. Several methods have been developed to monitor toxic metals in various sample matrices such as atomic absorption spectroscopy [16, 17], graphite furnace atomic absorption spectrometry [18, 19], inductively coupled plasma-atomic emission spectrometry [20]. These methods are sensitive; however, they require laborious sample preparation, skilled personnel, and high operational costs, which make them not suitable for fieldwork analysis. These impediments demand a simple, rapid and sensitive method for the determination of lead and cadmium.

Electrochemical methods primarily based on chemically modified solid electrodes are alternative methods because of its high sensitivity, selectivity, and suitability for fieldwork, and widely used for the determination of lead and cadmium [21, 22, 23]. Different chemically modified electrodes based on composite film of multiwall carbon nanotube with polyalizarin by forming complex formation with metals [24], bismuth-film composites [25, 26, 27], and bismuth nanoparticles [28] have attracted considerable attention because it extends to the negative potential limit. However, bismuth modified electrode works at higher concentration dynamic range and longer deposition time [25]. When *Nafion* polymer was added to bismuth film electrode the modified electrodes gave better results for the simultaneous determination of lead and cadmium [29, 30, 31, 32]. *Nafion* has been used as a protective layer to improve the detection limit and minimize the effect of fouling during electrochemical measurements [33]; however, it is an expensive material for routine sample analysis.

To overcome this limitation, a combination of polymer and bismuth-film modified electrodes has developed from cheaper monomers of different aminonaphthalenesulphonic acids such as 5-aminonaphthalene-1-sulphonic acid, 2-aminonaphthalene-1-sulphonic acid, 8-aminonaphthalene-2-sulphonic acid, and 4-amino-3-hydroxynaphthalene-1-sulphonic acid. These monomers were polymerized on glassy carbon electrode potentiodynamically followed by in-situ deposition of bismuth films. The incorporation of poly (8-aminonaphthalene-2-sulphonic acid) with bismuth modified electrode exhibits a new and cheaper approach for the determination of heavy metal ions without the use of the expensive *Nafion* polymer.

## 2 Experimental

### 2.1 Reagents and apparatus

Analytical grade acetic acid, sodium acetate, bismuth nitrate, cadmium nitrate, lead nitrate, hydrochloric acid from Sigma-Aldrich, and de-ionized water were used for solution preparation. Reagent grades 5AN1SA, 2AN1SA, 8AN2SA, and 4A3HN1SA from Sigma-Aldrich were used for the preparation of the polymer electrodes. All the electrochemical measurements were performed with a CHI 840C Electrochemical Workstation from CH Instruments (Austin, Texas, USA) in a one-compartment three-electrode system consisting of glassy carbon (GCE) working electrode, a platinum wire counter electrode, and an Ag/AgCl/Cl (3.0M) reference electrode. Digital JENWAY 3510 pH-meter was used to measure pH. Magnetic stirrers using a ceramic magnetic stirrer (JIJE LAB GLASS), ADAM electronic balance (model AE-437767) and BRANSONIC (ULTRASONIC CLEANER, model 2510E-DTH) were used whenever necessary.

### 2.2 Preparation of polymer-modified/GCE

Prior to electrochemical modification, GCE was polished up to the mirror finish using aqueous slurries of 0.05 μm alumina on a polishing pad, then sonicated successively with 1:1 nitric acid, ethanol, and distilled water for 5 min, respectively. Poly (5AN1SA), poly (2AN1SA)], poly (8-poly (8AN2SA)] and poly (4A3HN1SA) were electrodeposited on GCE following previously reported procedures [34]. Briefly, the respective polymers were deposited on a clean GCE by electropolymerization from of 2.0 mM of the monomers in 0.1 M HNO_3_ solution by potentiodynamically scanning the potential from −0.8 to +2.0 V for 15 cycles at 0.1 V/s. The polymer films on the electrodes were then stabilized in monomer free 0.5 M H_2_SO_4_ solution by scanning the potential between −0.8 and +0.8 V until the voltammogram become stable.

### 2.3 Analytical procedure

The experimental conditions were first optimized by varying the deposition potential (−0.6 – 1.6 V), deposition time (60–240 s), pH of the supporting electrolyte (ABS) (3.0–6.0), film thickness of the polymer electrodes (5–25 cycle) and the concentration of Bi(III) (0.1–2.5 mg/L). Then the determination of Pb(II) and Cd(II) in water samples was performed using Square Wave Anodic Stripping Voltammetry (SWASV) under the optimized conditions by scanning the potential range from −1.2 to 0.2 V. The potential step, square wave amplitude, and frequency were set to 4 mV, 0.025 mV, and 15 Hz, respectively. The calibration curves were used to determine the concentrations of Pb(II) and Cd(II) in wastewater samples. The interference study was carried out by measuring 30 μg/L of Pb(II) and Cd(II) in the presence of potential interfering ions with three fold higher concentration than the analyte amount. Recovery study was also performed by spiking 10, 20, and 30 μg/L of Pb(II) and Cd(II) in to the wastewater samples.

### 2.4 Computational details

The conformers of the dimers were generated using either the Marvin View [35] or PC Model [36] program packages employing the MMFF94 force field with a strict optimization limit. These conformers were used for further geometry optimization using the ωB97X-D functional [37] and the 6-31+G (d,p) basis sets [38] to identify the most stable conformers. The Pb(II) and Cd(II) ions were complexed with the optimized dimers after analyzing the natural charges obtained from the natural bond orbital (NBO) analysis. The geometries of the Pb(II) and Cd(II) complexes were then optimized using the ωB97X-D functional, 6-31+G (d,p) basis sets for the light atoms and the all-electron relativistic dyall-cvdz basis sets [39] for Pb(II) and Cd(II) to account for relativistic effects. The xyz coordinates of the optimized geometries of the dimers and their corresponding Cd(II) and Pb(II) complexes are presented in Table S-1. The optimized geometries were confirmed to be real minima on the potential energy surface with no imaginary frequencies by performing a vibrational analysis at the same level of theory. The changes in Gibbs free energies were all calculated at standard conditions (298.15 K and 1 atm). All the density functional theory (DFT) calculations were performed within a continuum solvent model by employing the polarizable continuum model (PCM) in its integral equation formalism variant (IEF-PCM) [40, 41]. Since the main aim of the study is for the application of the sensors in aqueous environments, we used water as a solvent in all the geometry optimizations, frequency calculations, and energy analyses. All the DFT calculations were performed using the Gaussian 09 program package [42]. Following the same approach as in other related studies [43, 44, 45], we define the interaction energy of the dimers and their corresponding metal ion complexes (Eq. 1):

(1) Δ*E*_int_(M@D) = *E*(M@D) - *E*(M) - *E*(D)

where the geometries of M (the metal ions) and D (the dimers) were the same as they are in the complex. To avoid basis sets superposition errors (BSSE), we used the Boys-Bernardicounterpoise scheme [46] where both molecules M and D were calculated in the M@D orbital basis set. To describe the stability of the complexes, we calculated the binding energies using Eq. (2):

(2) Δ*E*_bind_ = Δ*E*_int_(M@D) + Δ*E*_defom_(M) + Δ*E*_deform_(D)

where Δ*E*_int_(M@D) is the interaction energy for the M@D complex (Eq. 1), and Δ*E*_deform_(M) and Δ*E*_deform_(D) are the deformation energies for M and D, respectively (note that the deformation energy for M is zero). The deformation energies were calculated as the difference between the energy of M in the complex M@D orbital basis set and both M and D in their isolated states. The stabilization energies of the complexes, the change in zero point energy was added to the binding energy (Eq. 3):

(3) Δ*E*_stab_ = Δ*E*_bind_ + ΔE_ZPV_

## 3 Results and discussion

### 3.1 Electrosynthesis and characterization of the poly(ANSA) modified glassy carbon electrodes

**Figure 1:**
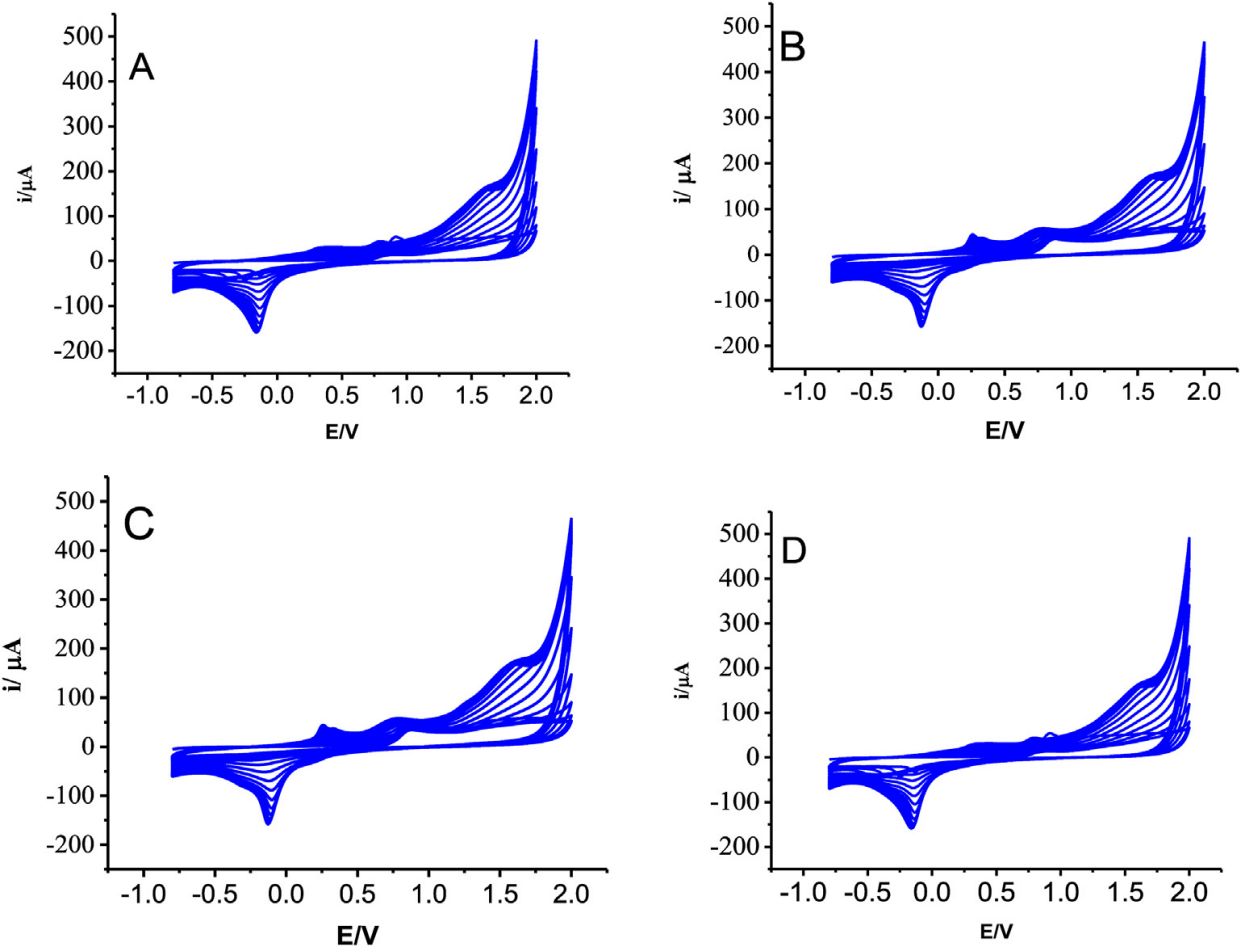
Typical cyclic voltammograms of 2.0 mM monomer: (A) 8AN2SA, (B) 5AN1SA, (C) 2AN1SA and (D) 4A3HN1SA in 0.1 M HNO_3_ for 15 cycles at scan rate of 0.1 V/s.

The polymer film electrodes were prepared on glassy carbon electrode by cycling the potential between -0.8 to +2.0 V for 15 cycles at 0.1 V/s from 2.0 mM monomer solutions prepared in 0.1 M HNO_3_. Typical cyclic voltammograms for the electrosynthesis of the polymer film are shown in Figure 1. The formation of the polymer film can be seen from the increasing peak currents near -0.11 V in the cathodic sweep and peaks of 0.28 V and 1.57 V in the anodic sweep. The observed distinct peaks during electropolymerization in Figure 1 and the corresponding activation at the polymer modified GCE demonstrate the deposition of electro-active polymer film on the surface, which are characteristics of these class of materials [34]. Moreover, nano-sized electroactive conductive films of about 40–60 nm are being formed by potentiodynamic method as reported in our previous work [47].

**Figure 2:**
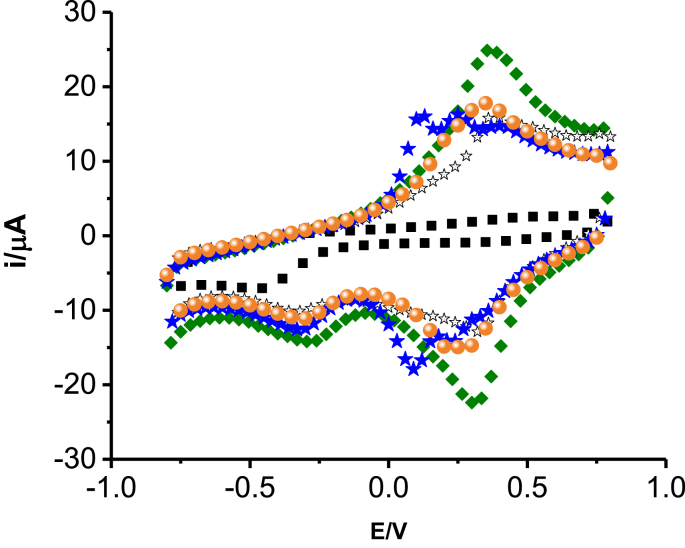
Cyclic voltammograms of monomer free () bare, () poly (8-AN2SA), () poly (4-A3HN1SA), () poly (2-AN1SA), () poly (5-AN1SA), at GCE in 0.5 M H_2_SO_4_ at scan rate 0.1 V/s.

The cyclic voltammograms (Figure 2) shows the electrochemical response of the bare GCE and poly (ANSA) modified GCE in a monomer free 0.5 M H_2_SO_4_. All polymer modified GCEs showed distinct peaks at 0.32 V and 0.34 V which was not shown in bare GCE. Thus, the results suggested an electroactive polymer film is deposited on the GCE. The highest peak current was observed for poly (8-AN2SA which suggest that it has better electrochemical response.

The mechanism of electron transfer at poly (8AN2SA)/Bi/GCE in monomer free of 0.5 M H_2_SO_4_ solution was evaluated using cyclic voltammetry for various scan rate in the range of 25–250 mV/s. The anodic and cathodic peak currents response on the polymer films was directly proportional to the scan rate (Fig. S-1**),** indicating that the reaction involves surface-controlled redox process [48]. Surface coverages, Γ, for different polyaminonaphthalene sulphonic acid derivates modified electrodes were calculated using Eq. 4 and Eq. 5 [49].

(4) $$i_{p}=\frac{n^{2}F^{2}}{4RT}\upsilon A\Gamma$$

(5) Q=nFAΓ

where n is the number of electrons transferred, F is the Faraday's constant, R is the gas constant, T is the temperature (K), A is the area of the electrode.

The calculated values were put in Table 1 and poly (8-AN2SA) shows the highest surface coverage (1.01 μmol/cm^2^), among other polymer films. In addition, it exhibits higher peak current response compared to other voltammograms as shown in Figure 2 and hence poly (8AN2SA) was chosen for further experiments.

**Table 1:** Surface coverage of the polymer modified GCE.

Polymer at GCE	Surface excess (μmol/cm^2^)
poly(8AN2SA)	1.01
poly(2AN1SA)	0.415
poly(5AN1SA)	0.108
poly(4A3HN1SA)	0.721

### 3.2 Computational investigations

The structures of the monomers used for the preparation of the polymer electrodes and computational calculations are shown in Fig. S-2 and Table 2, respectively. Structurally, the monomers are derivatives of the well-studied conducting polymer, polyaniline. The synthesis, characterization and applications of some of them are reported earlier by our group [34]. In our previous reports, we used these polymers for electrocatalytic reduction of oxygen [50]. Based on our previous experience and simplicity of the preparation of these polymer electrodes, we are now particularly interested to systematically investigate the application of these structurally similar to polyaniline derivatives for the detection of metal ions and select the best polymer electrode guided by computational calculations. Accordingly, computational investigations were carried out and the results are summarized in Table 2.

**Table 2:** Dimer deformation (ΔE_deform_), interaction (ΔE_int_), binding (ΔE_bind_), zero-point vibrational (ΔE_ZPVE_), and stabilization (ΔE_stab_) energies (all in kcal/mol), and total atomic charges of the metal ions in the complexes of the dimer-Pb(II) and dimer-Cd(II) complexes calculated using ωB97X-D/6-31+G (d,p)/dyall-cvdz/IEF-PCM/Water.

Complexes	Δ*E*_deform_	Δ*E*_int_	Δ*E*_bind_	Δ*E*_(ZPE)_	Δ*E*_stab_	Total atomic charges
Pb(II) complexes						
Pb(II) @dimer-2AN1SA	-25.66	-57.92	-83.58	2.74	-80.84	1.221
Pb(II) @dimer-4A3HN1SA	-19.45	-41.83	-61.29	0.02	-61.26	1.160
Pb(II) @dimer-5AN1SA	-24.47	-50.95	-75.42	1.17	-74.25	1.062
Pb(II) @dimer-8AN2SA	-15.54	-35.62	-51.16	0.62	-50.54	1.212
Cd(II) complexes						
Cd(II) @dimer-2AN1SA	-11.46	-8.60	-20.06	1.73	-18.33	1.831
Cd(II) @dimer-4A3HN1SA	-11.03	-3.22	-14.24	0.69	-13.56	2.001
Cd(II) @dimer-5AN1SA	-10.14	-4.21	-14.34	0.76	-13.59	1.987
Cd(II) @dimer-8AN2SA	-10.14	-3.21	-13.35	1.07	-12.28	1.997

The results indicate that the dimers (Fig. S-3) interestingly interact with Cd(II) and Pb(II) ions and form complex, as evidenced by the less exothermic energy. In particular, the results presented in the table show that the total energy for Pb(II) complexes are more exothermic than the Cd(II) complexes in general. The Cd(II)@dimer-8AN2SA complex showed relatively less binding energy (ΔE_bind_) with better stabilization energy compared with the other Pb(II) complexes. Moreover, the analysis of the total atomic charges listed in Table 1 show that Cd(II) ions in the complexes have an approximate charge of +2, whereas those of Pb(II) ions have a total atomic charges of approximately +1, indicating that the Pb(II) ions are interacting strongly with the polymers than Cd(II) ions. This is clear from the charge differences and the interaction energies of the complexes. Hence, under the same experimental conditions, Cd(II) is more sensitive than Pb(II) ions when complexed with the four polymers. From these we can deduce that the anodic stripping voltammetric signal of the Cd(II) complexes should be more intense than that of the Pb(II) complexes since the Cd(II) in the complexes are acting like free ions and need less energy to detach them from the polymer surface through oxidation. This could also be supported by the Hard and Soft Acids and Bases (HSAB) theory. According to this theory, Cd(II) is a soft acid whereas Pb(II) is an intermediate soft acid, indicating that Pb(II) ion can strongly bind with the polymer which has hard base functional group sites than Cd(II). On the other hand, Cd(II)@dimer-8AN2SA complex has relatively less binding energy (ΔE_bind_) and showed comparable stabilization energy with the other complexes listed in Table 2. Moreover, the deformation energy of the 8ANSA dimer is alsocomparably less than the other dimers, supporting the less binding energy of the complexes of this polymer. Thus, the calculated results predict that the polymer electrode made from the monomer 8AN2SA with the lowest binding and stabilization energy will be the best choice to get better anodic stripping voltammetric responses for both the Pb(II) and Cd(II). The differences will be expected to be more pronounced for Pb(II) than Cd(II).

### 3.3 Square wave anodic voltammetry study of poly(8AN2SA) modified films

**Figure 3:**
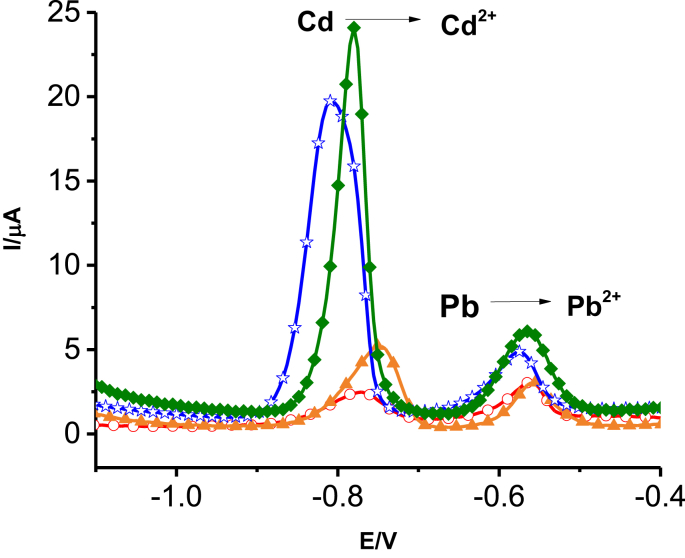
Anodic stripping square wave voltammograms of 50 μg/L of Cd(II) and Pb(II) on () bare GCE, () Bi/GCE, () poly (8AN2SA)/GCE and () poly (8AN2SA)/Bi/GCE in 0.1 MABS. The potential step, square wave amplitude and frequency were 4 mV, 0.025 mV and 15 Hz, respectively.

The SWASV responses of the in-situ deposited bismuth polymer at GC electrodes were compared under the same experimental conditions (Figure 3). The poly (8AN2SA)/Bi/GCE (Figure 3d) showed relatively higher peak current response than others. Electrodeposition of metal particles in conducting polymers layers are known to increase conductivity and other properties like electrocatalysis and sensing [51]. Hence, the increased current response of the metal ions in the polyminonaphetalene sulphonic acid derivatives is presumably due to the additional surface sites provided by the polymer nanoparticles to accumulate more metals during the electrodeposition which further enhances the current responses during the stripping.

### 3.4 Optimization of detection conditions

**Figure 4:**
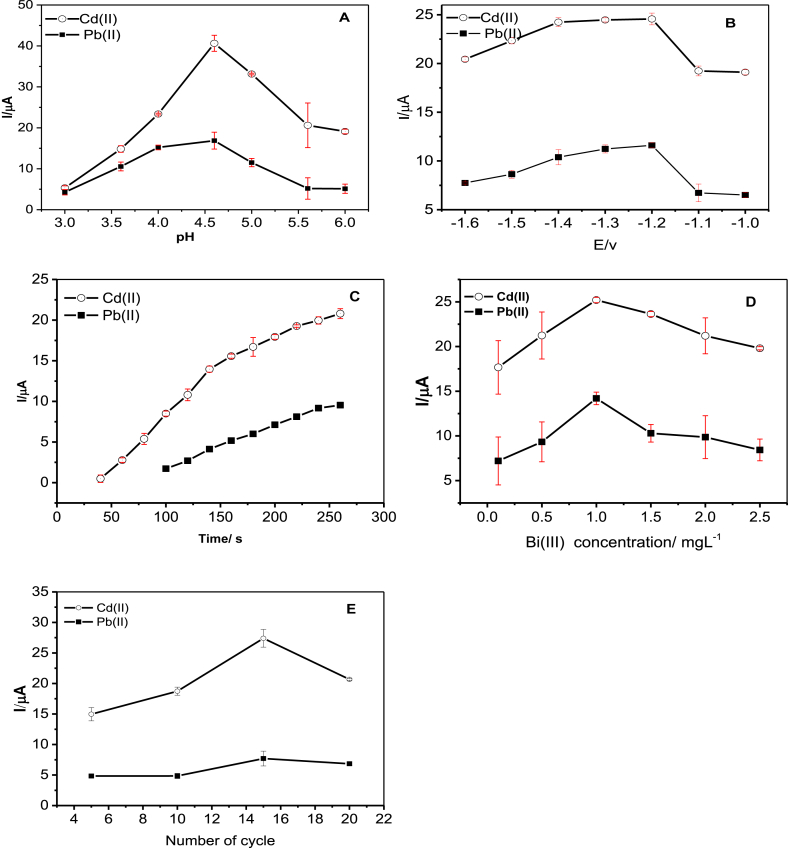
Effects of (A) pH, (B) deposition potential), (C) deposition time, (D) concentration of Bi(III, and (E) polymer film thickness on the stripping peak currents of 50 μg/L of Cd(II) and Pb(II) at Poly (8AN2SA)/Bi/GCE. Error bars are made from three replicate measurements (n = 3).

The effect of pH on the peak current of Pb(II) and Cd(II) was studied as shown in Figure 4A. The peak currents of Pb(II) and Cd(II) increased as the pH changed from 3 to 4.6, and then decreased with further increase of pH to 6.0. Low peak currents at lower pH can be attributed to the competition of hydrogen ions to analyte metal ions on the surface of the modified electrode. On the other hand, the decrease of peak current at higher pH may be due to the hydrolysis of both Cd(II) and Pb(II) ions [52]. Hence, 4.6 was the optimum pH for further experiments.

The effect of deposition potential on the peak current was investigated by changing the potential from -1.6 to -1.0 as shown in Figure 4B. Maximum peak current was observed at the deposition potential of -1.2 V for Pb(II) and -1.3 V and -1.2 V for Cd(II). However, deposition potential of -1.2 V has good repeatability and sensitivity was selected as the optimum potential for further experiments.

When the accumulation time was varied from 60 to 270 s, the peak current increased almost linearly with the increase of pre-concentration time (Figure 4C). There were a steep increment in peak current from 60 to 240 s but further increase of accumulation time results in a steady grow of peak currents. Thus by considering the high sensitivity as well as less analysis time, 240 s was selected as the optimal deposition time for further experiments.

Fig.4Dshows the effect of Bi(III) ion concentration on the peak currents of Pb(II) and Cd(II). Bi(III) ion is known to form fussed alloy with heavy metals and facilitate the nucleation process during the accumulation step. The result at low Bi(III) concentration is not favorable for efficient formation of Bi alloy [53]. Gradual decrease in peak current after 1 mg/L for both metal ions was observed. This effect was ascribed to the retarding effect of the thick bismuth film during mass transfer of metal ions in the stripping step [54].

The effect of poly (8AN2SA) film thickness on the peak current was also optimized by potentiodynamic electrodeposition (5,10,15 and 20 cycles) of the polymer films as shown in Figure 4E. Higher electroanyltical response was obtained for polymer film synthesized with 15 cycles. The lower peak current for the thin film may be due to the presence of a few active sites. For thicker films, a large background current was observed and sufficient sites may not be able to form the alloy with the target and bismuth metals.

### 3.5 Analytical performance

**Figure 5:**
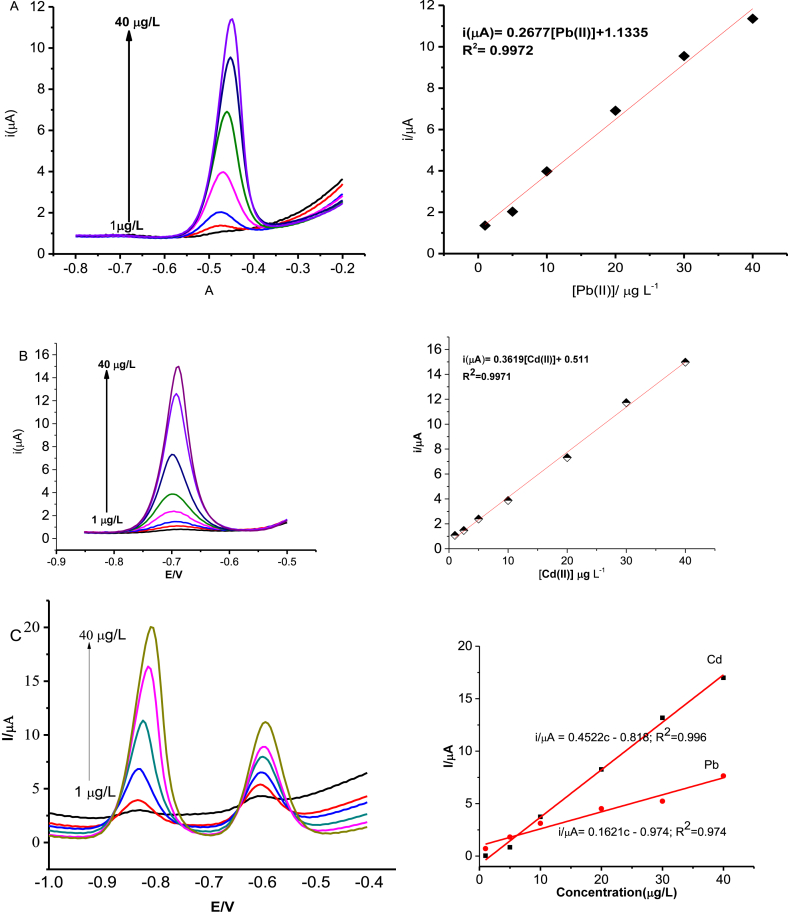
Anodic stripping square wave voltammograms of (A) Pb(II), (B) Cd(II), and (C) Cd(II) and Pb(II) ions in the concentration range of 1.0–40 μg/L at poly (8AN2SA)/Bi/GCE and their corresponding calibration curves.

The poly (8AN2SA)/Bi/GCE was used for the determination of Pb(II) and Cd(II) by applying SWASV and as shown in Figure 5. The anodic peak current was linearly increased in the range of 1.0 and 40.0 μg/L of Pb(II) with linear equation of i_p_ (μA) = 0.2677c + 0.079, correlation coefficient of R^2^ = 0.995 (Figure 5A). Similarly, a linear equation of i_p_ (μA) = 0.3619c - 0.804 with a correlation coefficient R^2^ = 0.992 was observed in a concentration range of 1.0–40.0 μg/L of Cd(II) ion (Figure 5B). The electrochemical response for the simultaneous determination of Pb(II) and Cd(II) at the poly (8AN2SA)/Bi/GCE was also performed (Figure 5C). The sensitivity of Cd(II) of the modified electrode is nearly three times than that of Pb(II) in agreement with the prediction of the theoretical calculations. Compared to Bi/GCE linear range of 40–200 μg/L and deposition time of 10 min [25], poly (8AN2SA)/Bi/GCE gave a lower linear range (1–40.0 μg/L) and lower deposition time (4 min). Moreover, detection limits for the simultaneous determination of Pb(II) and Cd(II) were found to be 0.38 and 0.08 μg/L, respectively, based on signal to noise ratio of 3. The results are compared with other modified electrodes reported in literatures as shown in Table 3.

**Table 3:** Comparison of different electrodes and methods for the detection of Pb(II) and Cd(II).

Electrode	Technique	Linear range (μg/L)		Detection limit (μg/L)		Reference
		Pb(II)	Cd(II)	Pb(II)	Cd(II)	
MWCNT-PARS/GCE	DPASV	5–150	5–160	0.47	0.43	[24]
BiFE/GCE	SWASV	40–200	40–200	0.30	-	[25]
Nafion/BiFE/GCE	DPASV	1.7–27.9	1.3–20.3	0.13	0.09	[26]
MWCNT/BiFE/GCE	SWASV	10–50	10–50	1.90	3.10	[27]
NanoBiE/GCE	ASV	5–60	5–60	0.80	0.40	[28]
Nafion/RGO-GNPs/BiFE/GCE	SWASV	1–90	1–90	0.08	0.12	[29]
Nafion/IL/Graphene/SPCE	SWASV	0.1–100	0.1–100	0.08	0.06	[30]
Nafion/BiF/GNFs/GCE	DPASV	0.2–50	0.2–50	0.02	0.09	[31]
Nafion/BiF/NMC/GCE	DPASV	0.5–100	2–100	0.05	1.50	[32]
Poy(8AN2SA)/Bi/GCE	SWASV	1–40	1–40	0.38	0.08	This work

### 3.6 Repeatability and interference study

**Figure 6:**
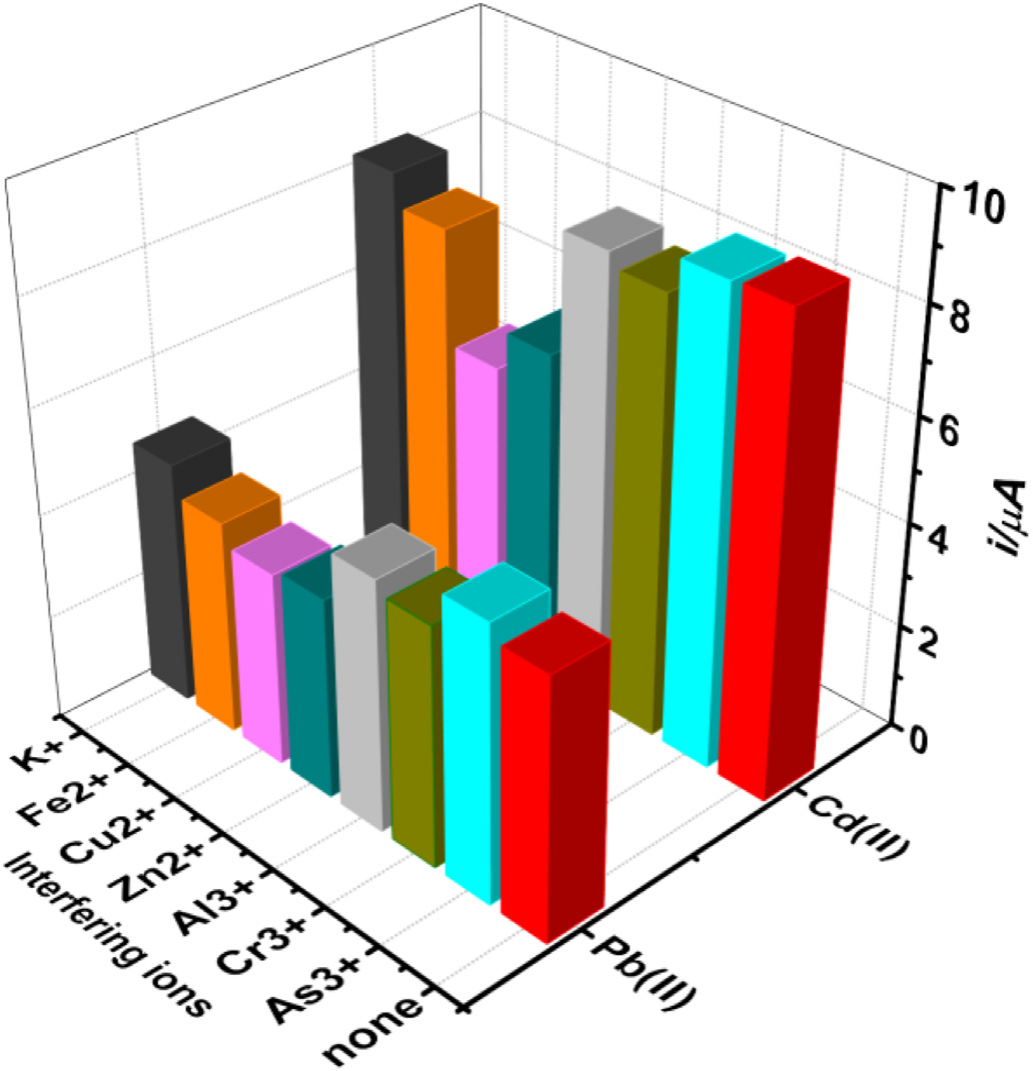
Effect of potentially interfering ions on the current response of 30 μg/L of Pb(II) and Cd(II) ions at poly (8AN2SA)/Bi/GCE.

Under the optimized condition, the stability of the poly (8AN2SA)/Bi/GCE was investigated by measuring anodic stripping responses of 30 μg/L of Pb(II) and Cd(II) ions for nine replicate measurements. The relative standard deviation (RSD) were found to be1.2% and 2.3% for Pb(II) and of Cd(II), respectively. The interference study was carried out by adding three fold higher concentration of potentially interfering ions (As^3+^, Cr^3+^, Al^3+^, Cu^2+^, Zn^2+^, Fe^2+^, and K^+^) into 0.1 M ABS containing 30 μg/L of Pb(II) and Cd(II). The anodic peak currents of both Pb(II) and Cd(II) ions decrease by less than 10% for most potentially interfering ions except for Cu(II) and Zn(II) as shown in Figure 6. The peak current of Pb(II)is lowered by nearly 31.0% in the presence of Zn(II) ion and 39% in the presence of Cu(II) while that of Cd(II) ion decreases nearly by 23.0% and 26.0% due to these ions, respectively. The interference of Zn(II) and Cu(II) on lead ion is more pronounced, this can be attributed the formation of intermetallic compounds and competition of these ions on the active sites present on the surface of the modified electrode [55].

### 3.7 Real sample analysis

The application and validity of the poly (8AN2SA)/Bi modified electrode for the determination of Pb(II) and Cd(II) were tested in the industrial wastewater sample collected (from Wonji Gefersa pulp Industry, East Showa, Ethiopia). The electrochemical result of Pb(II) in the wastewater was found to be 657.1 μg/L, which is comparable with the result from AAS (625.0 μg/L) with p-value 0.002. Similarly, the anodic stripping result in the wastewater for Cd(II) was found to be 93.8 μg/L, close to the result of AAS (96.6 μg/L) with p-value 0.04. The p-values in both cases are less than 0.05 which tells no significant difference between the two methods, indicating that the simple and cheap electrochemical method, anodic stripping voltammetry applied for the determination of Pb(II) and Cd(II) at poly (8AN2SA)/Bi modified glassy carbon electrode can be useful for the analysis of heavy metal ions.

Furthermore, in order to evaluate the reliability of the poly (8AN2SA)/Bi modified electrode, recovery studies were carried out by spiking standard sample to the industrial wastewater samples. It can be seen that the recoveries obtained were found to be 83.9–90.9% for Cd(II) and 92.0–101.0% for Pb(II) (see Table 4). The results reveal that the poly (8AN2SA)/Bi modified glassy carbon electrode can be used for the determination of Pb(II) and Cd(II) and has a good potential for the analysis of wastewater samples.

**Table 4:** Recovery tests in wastewater by spiking 10, 20 and 30 μg/L of Cd(II) and Pb(II).

Analyte	Detected (μg/L)	Added (μg/L)	Found (μg/L)	Recovery (%)
Cd(II)	3.61	10.0	12.7	90.9
		20.0	22.3	93.5
		30.0	35.5	106.3
Pb(II)	7.39	10.0	17.6	102.1
		20.0	27.6	101.1
		30.0	35.0	92.0

## 4 Conclusions

Among the several different aminonaphetalenesulphonic acid derivatives, poly (8-aminonaphthalene-2-sulphonic acid) was found to be the best polymer for in-situ modification bismuth film on glassy carbon electrode in agreement with the prediction from computational studies (DFT). For the simultaneous determination of Pb(II) and Cd(II), poly (8AN2SA)/bismuth film glassy carbon modified electrode exhibited a linear range of 1.0–40 μg/L with detection limits of 0.38 and 0.08 μg/L, respectively. Hence, the proposed method provides an alternative and cheaper modifier that can replace the expensive Nafion polymer.

## Declarations

### Author contribution statement

Alemayehu Yifru: Performed the experiments; Wrote the paper.

Gossa Dare: Performed the experiments.

Taye B. Demissie: Analyzed and interpreted the data; Wrote the paper.

Solomon Mehretie; Shimelis Admassie: Conceived and designed the experiments; Analyzed and interpreted the data; Wrote the paper.

### Funding statement

This research did not receive any specific grant from funding agencies in the public, commercial, or not-for-profit sectors.

### Data availability statement

Data will be made available on request.

### Declaration of interests statement

The authors declare no conflict of interest.

### Additional information

No additional information is available for this paper.
