# Supplementary material for: Cheap and sensitive polymer/bismuth film modified electrode for simultaneous determination of Pb(II) and Cd(II) ions
Source: Heliyon. 2021 Oct 19;7(10):e08215. doi: 10.1016/j.heliyon.2021.e08215 (PMC8648549; doi:10.1016/j.heliyon.2021.e08215)
Supplement: supporting information [file mmc1.docx]

**Supplementary material**

Cheap and sensitive polymer/bismuth film modified electrode for simultaneous determination of Pb(II) and Cd(II) ions

Alemayehu Yifru^a^, Gosa Dari^a^, Taye Beyene^b^,Solomon Meheretie^a^* and Shimelis Admassie^a,c^

1. Department of Chemistry, Addis Ababa University, PO Box 1176, Addis Ababa, Ethiopia
2. Materials Science Program, Department of Chemistry, Addis Ababa University, PO Box 1176, Addis Ababa, Ethiopia
3. State Key Laboratory of Luminescent Materials and Devices, Institute of Polymer Optoelectronic Materials and Devices, School of Materials Science and Engineering, South China University of Technology, 381 Wushan Road, Guangzhou, P.R. China.


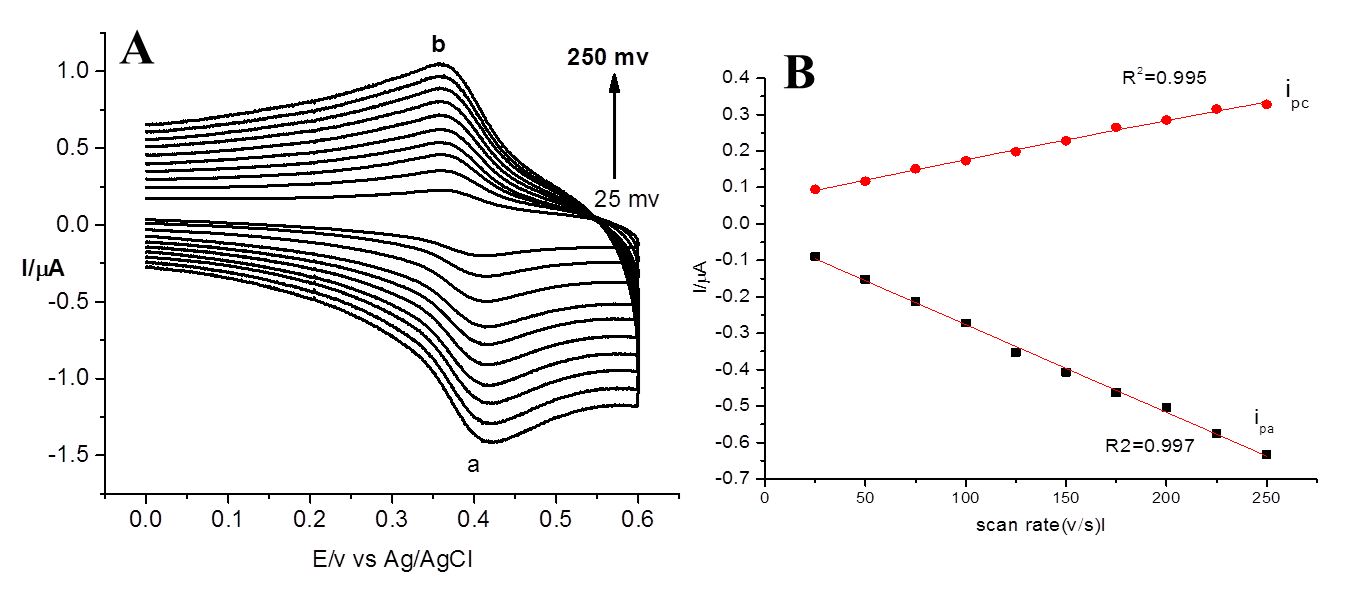


**Fig. S-****1** Cyclic voltammograms of poly(8-ANSA )/GCE in 0.5 M H_2_SO_4_ at various scan rate 25, 50, 75, 100, 125, 150, 175, 200, 225 and 250 mV/s.

 ****

(a) (b) (c) (d)

**Fig. S-2** Chemical structures of (a) 8-aminonaphthalene-2-sulphonic acid (8AN2SA), (b) 5-aminonaphthalene-1-sulphonic acid (5AN1SA), (c) 2-aminonaphthalene-1-sulphonic acid (2AN1SA), and (d) 4-amino-3-hydroxynaphthalene-1-sulphonic acid (4A3HN1SA).

**Fig. S-3** Dimers of (a) 2-ANSA, (b) 4-ANSA, (c) 5-ANSA, and (d) 8-ANSA molecular cluster. Hydrogen atoms were omitted for clarity

**Table S­-1:** Optimized geometries of the dimers and the corresponding metal complexes

**2AN1SA**, ωB97XD/6-31+G(d,p)/Dyall-cvdz, in water

*G* = -1504.522385 hartree, HF = -1504.798156 hartree

C 2.733553000 -0.575317000 -0.084228000

C 2.463812000 -1.970382000 -0.107716000

C 1.135121000 -2.421640000 0.137034000

C 0.121090000 -1.557403000 0.390145000

C 0.332222000 -0.139648000 0.423997000

C 1.636074000 0.331792000 0.182734000

C 4.071988000 -0.172863000 -0.338374000

C 5.059467000 -1.099057000 -0.595736000

C 4.781305000 -2.478858000 -0.615567000

C 3.496133000 -2.900543000 -0.371976000

C -4.213603000 -0.370062000 -0.339696000

C -2.868891000 0.087259000 -0.423195000

C -2.350744000 0.450325000 -1.695452000

C -3.135342000 0.363659000 -2.821604000

C -4.473497000 -0.087004000 -2.737832000

C -4.997957000 -0.445628000 -1.518733000

C -4.728562000 -0.741437000 0.934143000

C -3.957926000 -0.669322000 2.059960000

C -2.608979000 -0.211299000 1.994353000

C -2.086473000 0.160391000 0.766089000

N -0.748192000 0.646666000 0.706238000

S 1.987450000 2.053516000 0.188322000

N -1.828275000 -0.209472000 3.135419000

O 3.143923000 2.387120000 1.011325000

O 0.775401000 2.837463000 0.417649000

O 2.416442000 2.259126000 -1.358650000

H 0.935867000 -3.489155000 0.119177000

H -0.880605000 -1.926278000 0.574730000

H 4.345404000 0.872564000 -0.322452000

H 6.070288000 -0.751192000 -0.784188000

H 5.570476000 -3.194722000 -0.818758000

H 3.249600000 -3.958456000 -0.379905000

H -1.325564000 0.798554000 -1.771940000

H -2.723783000 0.644216000 -3.786322000

H -5.080396000 -0.148742000 -3.635302000

H -6.024345000 -0.794378000 -1.441851000

H -5.754956000 -1.090429000 1.004287000

H -4.364724000 -0.962544000 3.023252000

H -0.615487000 1.648619000 0.646772000

H -2.322373000 -0.140849000 4.013155000

H -1.002686000 0.372464000 3.094305000

H 3.020474000 3.016274000 -1.432365000

**4A3HN1SA,** ωB97XD/6-31+G(d,p)/Dyall-cvdz, in water

*G* = -1654.923330 hartree, HF = -1655.205168 hartree

C -4.348598000 0.177271000 -0.207271000

C -3.331128000 -0.722176000 0.235424000

C -1.963295000 -0.413100000 -0.047462000

C -1.659650000 0.713284000 -0.774889000

C -2.679482000 1.564193000 -1.248662000

C -4.010927000 1.325502000 -0.983667000

C -5.699029000 -0.107506000 0.136614000

C -6.033451000 -1.235650000 0.845211000

C -5.031316000 -2.148535000 1.241643000

C -3.714780000 -1.892108000 0.944001000

C 2.461682000 0.279030000 0.865272000

C 1.085397000 -0.020020000 1.087710000

C 0.403493000 0.624126000 2.153907000

C 1.056041000 1.516047000 2.966735000

C 2.421764000 1.807359000 2.752517000

C 3.108128000 1.204986000 1.728023000

C 3.100324000 -0.377163000 -0.233864000

C 2.444416000 -1.253588000 -1.055641000

C 1.084264000 -1.543731000 -0.810299000

C 0.414891000 -0.943225000 0.233480000

N -0.952607000 -1.300036000 0.397602000

O -2.385210000 2.681014000 -1.983816000

N -4.996109000 2.234282000 -1.414523000

O 0.468348000 -2.419998000 -1.628937000

S 4.814193000 -0.009556000 -0.566195000

O 5.629664000 -0.416558000 0.568397000

O 4.923496000 1.344635000 -1.076516000

O 5.167528000 -0.961422000 -1.810309000

H -0.625550000 0.961512000 -0.995447000

H -6.481407000 0.586226000 -0.152139000

H -7.071223000 -1.427847000 1.098527000

H -5.300390000 -3.050786000 1.780964000

H -2.963862000 -2.614745000 1.245727000

H -0.647939000 0.411915000 2.315500000

H 0.521474000 2.003180000 3.775416000

H 2.931400000 2.513072000 3.399607000

H 4.155219000 1.444863000 1.584791000

H 2.939501000 -1.734879000 -1.889369000

H -1.143948000 -1.713047000 1.301998000

H -1.437459000 2.744594000 -2.138754000

H -4.623831000 2.900428000 -2.080240000

H -5.814122000 1.789184000 -1.812715000

H -0.430981000 -2.541637000 -1.278452000

H 5.523982000 -1.810225000 -1.501088000

**5AN1SA**, ωB97XD/6-31+G(d,p)/Dyall-cvdz, in water

*G* = -1504.517598 hartree, HF = -1504.792426 hartree

C 4.627248000 -0.026304000 0.143095000

C 3.292172000 0.026043000 -0.357716000

C 2.478201000 -1.143980000 -0.240436000

C 2.952844000 -2.275502000 0.375334000

C 4.259202000 -2.304872000 0.908343000

C 5.077242000 -1.208537000 0.788748000

C 5.471536000 1.127618000 -0.003594000

C 4.956174000 2.274522000 -0.578361000

C 3.622704000 2.321305000 -1.031639000

C 2.800994000 1.225325000 -0.938328000

C -1.226829000 -0.609951000 -0.594095000

C -2.320816000 -0.110372000 0.179932000

C -3.589661000 -0.036416000 -0.479241000

C -3.779118000 -0.440559000 -1.779142000

C -2.694370000 -0.957306000 -2.511258000

C -1.455311000 -1.038288000 -1.927305000

C 0.081526000 -0.685224000 0.003265000

C 0.239742000 -0.328856000 1.329688000

C -0.856118000 0.131425000 2.080883000

C -2.108706000 0.257668000 1.533495000

N 1.149223000 -1.148660000 -0.760577000

N 6.773775000 1.102784000 0.492325000

S -5.023385000 0.608434000 0.351190000

O -6.172201000 0.496231000 -0.533867000

O -4.726063000 1.883558000 0.978599000

O -5.240288000 -0.396410000 1.595648000

H 2.305951000 -3.143565000 0.451236000

H 4.615046000 -3.199137000 1.408866000

H 6.073481000 -1.251114000 1.215304000

H 5.589915000 3.150313000 -0.685228000

H 3.249114000 3.241862000 -1.469544000

H 1.777814000 1.276995000 -1.293234000

H -4.761082000 -0.369228000 -2.231503000

H -2.846499000 -1.296147000 -3.529325000

H -0.645360000 -1.466737000 -2.506720000

H 1.217803000 -0.389609000 1.792875000

H -0.697026000 0.409233000 3.118043000

H -2.921728000 0.637353000 2.136925000

H 1.100494000 -0.932661000 -1.746098000

H 7.348700000 1.872640000 0.178144000

H 7.268246000 0.227192000 0.392542000

H -5.625207000 -1.234668000 1.293377000

**8AN2SA**, ωB97XD/6-31+G(d,p)/Dyall-cvdz, in water

*G* = -1504.522903 hartree, HF = -1504.797676 hartree

C 4.109451000 -0.571945000 0.180838000

C 4.556914000 0.210642000 -0.920429000

C 3.600076000 0.995013000 -1.623252000

C 2.277915000 0.999991000 -1.272484000

C 1.828984000 0.218300000 -0.173278000

C 2.732860000 -0.544744000 0.532718000

C 5.068366000 -1.356861000 0.905699000

C 6.388253000 -1.359814000 0.494308000

C 6.811796000 -0.596757000 -0.614945000

C 5.921796000 0.184348000 -1.309432000

C -2.628554000 2.247259000 0.418802000

C -1.778850000 1.120704000 0.217469000

C -0.354754000 1.294059000 0.274358000

C 0.160502000 2.559126000 0.474321000

C -0.692895000 3.671391000 0.633401000

C -2.058031000 3.528889000 0.622103000

C -4.039935000 2.064373000 0.391155000

C -4.595832000 0.836917000 0.152830000

C -3.733282000 -0.260582000 -0.078662000

C -2.366944000 -0.139513000 -0.052634000

N 4.648095000 -2.156825000 1.972524000

N 0.455754000 0.160432000 0.124895000

S -4.443207000 -1.844876000 -0.420140000

O -3.396885000 -2.768029000 -0.830488000

O -5.636129000 -1.680861000 -1.230351000

O -5.015752000 -2.322459000 1.012266000

H 3.934198000 1.586838000 -2.470790000

H 1.561440000 1.585006000 -1.839841000

H 2.367409000 -1.148914000 1.357219000

H 7.114057000 -1.955964000 1.040694000

H 7.857349000 -0.626133000 -0.905857000

H 6.243992000 0.782438000 -2.156642000

H 1.233701000 2.702317000 0.530846000

H -0.250537000 4.649932000 0.790386000

H -2.709746000 4.384788000 0.764795000

H -4.680388000 2.925178000 0.555503000

H -5.671780000 0.707121000 0.124950000

H -1.747891000 -1.002571000 -0.267499000

H 3.945859000 -1.745386000 2.572529000

H 5.409228000 -2.521001000 2.530033000

H 0.194901000 -0.623828000 0.707148000

H -4.294309000 -2.641056000 1.578088000

**Pb(II)@dimer-2AN1SA**, ωB97XD/6-31+G(d,p)/Dyall-cvdz, in water

*G* = -21033.248291 hartree, HF = -21033.52572 hartree

C -3.040210000 0.199114000 0.571625000

C -2.907178000 0.407220000 1.975161000

C -1.616722000 0.501330000 2.546318000

C -0.491383000 0.386633000 1.781035000

C -0.585332000 0.174151000 0.387766000

C -1.837564000 0.092933000 -0.214364000

C -4.357187000 0.114525000 0.040835000

C -5.454505000 0.230409000 0.859689000

C -5.315103000 0.435942000 2.250022000

C -4.060654000 0.521272000 2.794473000

C 2.683582000 3.152253000 0.248559000

C 1.629115000 2.297017000 -0.181035000

C 0.519350000 2.872119000 -0.854821000

C 0.459169000 4.226655000 -1.074459000

C 1.500107000 5.076630000 -0.636691000

C 2.590351000 4.547736000 0.007213000

C 3.813922000 2.597971000 0.905637000

C 3.904619000 1.253365000 1.132180000

C 2.856180000 0.399840000 0.718749000

C 1.743969000 0.897176000 0.087028000

N 0.674656000 -0.008007000 -0.315693000

S -2.015372000 -0.097466000 -1.978388000

N 2.974291000 -1.026343000 0.992029000

O -2.920757000 -1.174388000 -2.328671000

O -0.692690000 -0.100157000 -2.603014000

O -2.710409000 1.311698000 -2.291640000

H -1.522870000 0.665750000 3.614802000

H 0.485157000 0.472773000 2.242320000

H -4.519722000 -0.056698000 -1.013595000

H -6.446259000 0.158855000 0.425634000

H -6.195671000 0.522875000 2.877066000

H -3.925500000 0.678193000 3.860066000

H -0.293327000 2.254344000 -1.213962000

H -0.397081000 4.646694000 -1.591439000

H 1.435238000 6.144533000 -0.815704000

H 3.400158000 5.189006000 0.341419000

H 4.613676000 3.257544000 1.226739000

H 4.768548000 0.828607000 1.631568000

H 0.508181000 0.080808000 -1.327678000

H 2.956842000 -1.185235000 2.003576000

H 3.899268000 -1.347857000 0.695868000

H -3.220865000 1.271033000 -3.118479000

Pb 1.335869000 -2.407125000 -0.063430000

**Pb(II)@dimer-4A3HN1SA**, ωB97XD/6-31+G(d,p)/Dyall-cvdz, in water

*G* = -21183.639341 hartree, HF = -21183.91698 hartree

C 3.567789000 1.102880000 0.366460000

C 2.432685000 1.373094000 -0.475131000

C 1.104262000 1.072444000 0.007988000

C 0.978472000 0.379125000 1.219339000

C 2.107879000 0.111384000 2.041844000

C 3.407173000 0.455469000 1.640706000

C 4.855705000 1.490094000 -0.096738000

C 5.017443000 2.088561000 -1.318020000

C 3.905062000 2.318756000 -2.162030000

C 2.647083000 1.973012000 -1.751544000

C -3.668181000 1.098808000 0.029286000

C -2.370555000 1.668451000 -0.122008000

C -2.186761000 3.052346000 0.131429000

C -3.248764000 3.843206000 0.491763000

C -4.539859000 3.285484000 0.620822000

C -4.745792000 1.946300000 0.401308000

C -3.794868000 -0.307915000 -0.184984000

C -2.728682000 -1.096742000 -0.524466000

C -1.466809000 -0.503982000 -0.723207000

C -1.274966000 0.844135000 -0.523011000

N -0.000772000 1.406987000 -0.730945000

O 1.992051000 -0.455356000 3.255230000

N 4.449333000 0.261965000 2.508374000

O -0.395974000 -1.276104000 -1.116304000

S -5.391497000 -1.073803000 0.046149000

O -6.338778000 -0.509519000 -0.901710000

O -5.708889000 -1.086245000 1.460979000

O -5.122900000 -2.605270000 -0.348166000

H -0.009912000 0.150026000 1.606590000

H 5.725495000 1.331298000 0.528282000

H 6.008850000 2.382483000 -1.644262000

H 4.050552000 2.768932000 -3.137218000

H 1.811167000 2.138245000 -2.420819000

H -1.193874000 3.479825000 0.050198000

H -3.095715000 4.899524000 0.684854000

H -5.374609000 3.917769000 0.903283000

H -5.744246000 1.540603000 0.513051000

H -2.846268000 -2.165374000 -0.660119000

H 0.079651000 2.146827000 -1.411908000

H 1.076677000 -0.654910000 3.484388000

H 4.248038000 -0.327352000 3.304292000

H 5.369100000 0.106414000 2.124293000

H -0.700510000 -2.088704000 -1.543794000

H -5.319308000 -2.759955000 -1.286851000

Pb 2.142012000 -1.476549000 -0.335289000

**Pb(II)@dimer-5AN1SA**, ωB97XD/6-31+G(d,p)/Dyall-cvdz in water, in water

*G* = -21033.236326 hartree, HF = -21033.51078 hartree

C 4.107877000 0.995550000 -0.164082000

C 2.780692000 1.070273000 0.326586000

C 1.941872000 2.101260000 -0.167598000

C 2.397108000 3.023905000 -1.081653000

C 3.718745000 2.950219000 -1.552826000

C 4.552689000 1.950156000 -1.107644000

C 4.990371000 -0.031082000 0.365922000

C 4.540788000 -0.864213000 1.417756000

C 3.257496000 -0.756919000 1.893595000

C 2.297966000 0.110846000 1.303006000

C -1.681757000 1.313688000 0.456513000

C -2.721659000 0.560380000 -0.159673000

C -3.999522000 0.608848000 0.464184000

C -4.235820000 1.334276000 1.612307000

C -3.189746000 2.047401000 2.215973000

C -1.939383000 2.033746000 1.645618000

C -0.367345000 1.327849000 -0.158974000

C -0.100755000 0.440885000 -1.229204000

C -1.200977000 -0.238249000 -1.861728000

C -2.457560000 -0.192999000 -1.353024000

N 0.594525000 2.156380000 0.297002000

N 6.228141000 -0.186901000 -0.103413000

S -5.386225000 -0.303912000 -0.186947000

O -6.561177000 0.011666000 0.606288000

O -5.013734000 -1.686886000 -0.415096000

O -5.578769000 0.302480000 -1.665257000

H 1.725571000 3.799891000 -1.433195000

H 4.077551000 3.680580000 -2.268398000

H 5.567067000 1.925723000 -1.488240000

H 5.228692000 -1.579228000 1.854234000

H 2.950906000 -1.380466000 2.728666000

H 1.448843000 0.407677000 1.921002000

H -5.226804000 1.343117000 2.050695000

H -3.370139000 2.597743000 3.131407000

H -1.147093000 2.573672000 2.151390000

H 0.787084000 0.630824000 -1.834070000

H -0.998709000 -0.807210000 -2.763327000

H -3.253389000 -0.730990000 -1.847974000

H 0.378508000 2.835665000 1.011237000

H 6.833040000 -0.896852000 0.281150000

H 6.603772000 0.360752000 -0.860152000

H -6.023141000 1.165060000 -1.630685000

Pb 1.023441000 -1.616862000 -0.058116000

**Pb(II)@dimer-8AN2SA**, ωB97XD/6-31+G(d,p)/Dyall-cvdz, in water

*G* = -21033.235695 hartree, HF = -21033.50583 hartree

C 3.143083000 0.509452000 0.760049000

C 3.377039000 1.032043000 -0.558787000

C 2.259912000 1.616093000 -1.241364000

C 1.025048000 1.776731000 -0.650940000

C 0.811699000 1.380742000 0.717175000

C 1.819339000 0.594675000 1.321996000

C 4.230104000 -0.120702000 1.459908000

C 5.462835000 -0.184812000 0.829643000

C 5.672000000 0.339744000 -0.459567000

C 4.654566000 0.942238000 -1.158567000

C -3.660552000 2.285475000 -0.282888000

C -2.584465000 1.591736000 0.339513000

C -1.447932000 2.341752000 0.763740000

C -1.398216000 3.702537000 0.595643000

C -2.473944000 4.384278000 -0.019440000

C -3.577265000 3.693135000 -0.450903000

C -4.792155000 1.551624000 -0.734931000

C -4.860179000 0.192335000 -0.585550000

C -3.783405000 -0.473415000 0.046891000

C -2.675074000 0.187054000 0.508441000

N 4.015676000 -0.684607000 2.701745000

N -0.341102000 1.659017000 1.356319000

S -3.867513000 -2.232589000 0.252992000

O -2.589553000 -2.719713000 0.747012000

O -4.475500000 -2.825539000 -0.922906000

O -4.977126000 -2.425371000 1.405572000

H 2.393765000 1.943654000 -2.267347000

H 0.221321000 2.243046000 -1.208225000

H 1.631482000 0.202799000 2.315184000

H 6.295709000 -0.640879000 1.355441000

H 6.661003000 0.266282000 -0.898236000

H 4.806118000 1.344836000 -2.153278000

H -0.524494000 4.250773000 0.932349000

H -2.416047000 5.459490000 -0.147287000

H -4.401742000 4.214070000 -0.927489000

H -5.606739000 2.086418000 -1.212607000

H -5.717599000 -0.366101000 -0.943701000

H -1.885767000 -0.364632000 1.004723000

H 3.398703000 -0.195611000 3.335098000

H 4.849854000 -1.002120000 3.175743000

H -0.439893000 1.336782000 2.310682000

H -4.599560000 -2.247291000 2.281980000

Pb 1.470913000 -1.096613000 -0.688356000

**Cd(II)@dimer-2AN1SA**, ωB97XD/6-31+G(d,p)/Dyall-cvdz, in water

*G* = -6972.008400 hartree, HF = -6972.282405 hartree

C -2.866548000 0.435038000 0.614180000

C -2.501191000 -0.024235000 1.908962000

C -1.124668000 -0.229391000 2.205922000

C -0.151757000 0.004210000 1.288164000

C -0.462045000 0.463612000 -0.033540000

C -1.814571000 0.678732000 -0.350252000

C -4.252002000 0.627527000 0.364564000

C -5.193470000 0.380811000 1.340143000

C -4.820278000 -0.071653000 2.620727000

C -3.488154000 -0.270681000 2.892955000

C 4.022188000 1.307885000 0.475598000

C 2.660703000 1.521702000 0.123926000

C 2.052940000 2.764774000 0.442291000

C 2.772669000 3.747858000 1.078265000

C 4.128683000 3.538830000 1.426650000

C 4.738559000 2.344057000 1.130962000

C 4.627494000 0.060714000 0.160144000

C 3.924051000 -0.926781000 -0.475230000

C 2.569200000 -0.716147000 -0.832151000

C 1.945563000 0.474629000 -0.530535000

N 0.583090000 0.650448000 -0.897950000

S -2.286814000 1.241638000 -1.948603000

N 1.841560000 -1.764896000 -1.468780000

O -3.340690000 0.424427000 -2.536950000

O -1.112593000 1.494907000 -2.781075000

O -2.932470000 2.672537000 -1.565739000

H -0.850851000 -0.576677000 3.197932000

H 0.888219000 -0.158324000 1.545152000

H -4.597237000 0.961180000 -0.603623000

H -6.242527000 0.539381000 1.110546000

H -5.574872000 -0.259699000 3.376968000

H -3.167876000 -0.619040000 3.870847000

H 1.014610000 2.933130000 0.175522000

H 2.298482000 4.695053000 1.315280000

H 4.683004000 4.325948000 1.927443000

H 5.778878000 2.175179000 1.394382000

H 5.666667000 -0.103250000 0.429491000

H 4.395914000 -1.875276000 -0.712521000

H 0.379875000 1.092651000 -1.786895000

H 2.445010000 -2.332170000 -2.057455000

H 1.096590000 -1.385469000 -2.049191000

H -3.605147000 2.916466000 -2.222639000

Cd 0.697699000 -3.287460000 -0.025143000

**Cd(II)@dimer-4A3HN1SA**, ωB97XD/6-31+G(d,p)/Dyall-cvdz, in water

*G* = -7122.402481 hartree, HF = -7122.681526 hartree

C 3.983166000 0.989768000 0.148176000

C 2.928317000 1.182657000 -0.794417000

C 1.575925000 1.001548000 -0.368173000

C 1.318506000 0.609467000 0.924772000

C 2.373435000 0.360212000 1.827698000

C 3.694098000 0.543644000 1.473258000

C 5.318759000 1.240663000 -0.272223000

C 5.601056000 1.621209000 -1.562050000

C 4.559885000 1.760881000 -2.507225000

C 3.256491000 1.548922000 -2.127120000

C -2.994328000 1.272885000 0.078198000

C -1.669484000 1.705289000 -0.227430000

C -1.236420000 2.978961000 0.228328000

C -2.079058000 3.792305000 0.942984000

C -3.395027000 3.369843000 1.238104000

C -3.841702000 2.141409000 0.818845000

C -3.375478000 -0.028739000 -0.376013000

C -2.526653000 -0.846983000 -1.073989000

C -1.226463000 -0.392274000 -1.378652000

C -0.798306000 0.850725000 -0.962951000

N 0.533920000 1.199840000 -1.311614000

O 2.128822000 -0.059572000 3.107584000

N 4.714324000 0.358582000 2.421505000

O -0.418782000 -1.207565000 -2.088389000

S -5.008744000 -0.628516000 0.016929000

O -6.008905000 0.249352000 -0.571806000

O -5.076470000 -0.941178000 1.432845000

O -5.076975000 -2.046371000 -0.733896000

H 0.295826000 0.476957000 1.265115000

H 6.131261000 1.147898000 0.440756000

H 6.628118000 1.812179000 -1.857020000

H 4.789655000 2.036746000 -3.531346000

H 2.467322000 1.647932000 -2.865037000

H -0.222136000 3.300073000 0.015775000

H -1.733951000 4.761608000 1.287313000

H -4.056351000 4.018294000 1.802831000

H -4.854994000 1.841266000 1.058977000

H -2.828341000 -1.831683000 -1.408031000

H 0.606514000 2.069226000 -1.826980000

H 1.183155000 -0.153769000 3.259609000

H 4.378178000 -0.104489000 3.256590000

H 5.526866000 -0.129833000 2.066384000

H 0.388297000 -0.697911000 -2.276588000

H -5.436112000 -1.941080000 -1.630092000

Cd 1.603272000 -2.943287000 0.157296000

**Cd(II)@dimer-5AN1SA**, ωB97XD/6-31+G(d,p)/Dyall-cvdz, in water

*G* = -6971.999074 hartree, HF = -6972.271774 hartree

C -4.405764000 -0.758194000 -0.068201000

C -3.064542000 -0.935715000 0.383729000

C -2.305833000 -2.022753000 -0.152600000

C -2.844648000 -2.866683000 -1.091209000

C -4.163389000 -2.668385000 -1.555169000

C -4.923343000 -1.639380000 -1.055321000

C -5.188464000 0.317035000 0.478886000

C -4.609841000 1.178345000 1.393641000

C -3.275478000 1.002193000 1.810476000

C -2.511287000 -0.035158000 1.332496000

C 1.371757000 -1.487133000 0.353798000

C 2.426370000 -0.704908000 -0.214909000

C 3.682936000 -0.727693000 0.471608000

C 3.898513000 -1.470102000 1.607995000

C 2.853485000 -2.250791000 2.136249000

C 1.627371000 -2.254948000 1.519979000

C 0.076066000 -1.483494000 -0.275916000

C -0.105769000 -0.760934000 -1.442388000

C 0.954958000 -0.026573000 -2.001533000

C 2.192816000 0.024421000 -1.408960000

N -0.959431000 -2.228590000 0.278125000

N -6.493679000 0.521719000 0.037965000

S 5.063365000 0.233810000 -0.102380000

O 6.214001000 -0.038554000 0.744772000

O 4.665827000 1.608980000 -0.346820000

O 5.363544000 -0.352132000 -1.574965000

H -2.238229000 -3.678616000 -1.479583000

H -4.572170000 -3.331310000 -2.310401000

H -5.926843000 -1.497428000 -1.442038000

H -5.196224000 1.995472000 1.804115000

H -2.855096000 1.695444000 2.532861000

H -1.488808000 -0.160855000 1.671086000

H 4.869794000 -1.458192000 2.088296000

H 3.025723000 -2.849062000 3.023393000

H 0.853160000 -2.886153000 1.941609000

H -1.073540000 -0.758285000 -1.930541000

H 0.779398000 0.525787000 -2.919482000

H 2.978295000 0.617304000 -1.857238000

H -0.880388000 -2.418530000 1.266432000

H -7.025906000 1.166000000 0.606936000

H -7.033135000 -0.310430000 -0.155543000

H 5.808839000 -1.212594000 -1.516546000

Cd -0.602696000 2.717653000 -0.121202000

**Cd(II)@dimer-8AN2SA**, ωB97XD/6-31+G(d,p)/Dyall-cvdz, in water

*G* = -6972.003102 hartree, HF = -6972.275426 hartree

C -3.175414000 -1.609048000 0.165568000

C -3.658513000 -1.262446000 1.458985000

C -2.768693000 -0.613760000 2.360648000

C -1.476642000 -0.326639000 2.013297000

C -0.994647000 -0.665090000 0.719918000

C -1.831210000 -1.295565000 -0.175532000

C -4.066761000 -2.254341000 -0.756481000

C -5.356277000 -2.550412000 -0.353354000

C -5.813501000 -2.220064000 0.940349000

C -4.990244000 -1.580025000 1.833954000

C 3.062763000 2.142413000 0.424291000

C 2.405197000 0.878480000 0.367849000

C 0.978076000 0.817870000 0.529018000

C 0.286686000 1.981738000 0.806729000

C 0.958023000 3.219759000 0.897370000

C 2.311958000 3.314352000 0.694165000

C 4.471214000 2.200892000 0.227488000

C 5.209818000 1.068827000 0.012134000

C 4.542792000 -0.178603000 0.000174000

C 3.185322000 -0.287840000 0.171381000

N -3.606374000 -2.633086000 -2.019922000

N 0.353556000 -0.428277000 0.392898000

S 5.493419000 -1.649971000 -0.248945000

O 4.666264000 -2.815275000 0.022305000

O 6.789926000 -1.506517000 0.386795000

O 5.832942000 -1.618622000 -1.827412000

H -3.128356000 -0.359985000 3.353946000

H -0.807390000 0.144369000 2.725933000

H -1.436759000 -1.563786000 -1.150611000

H -6.030691000 -3.042721000 -1.048728000

H -6.832827000 -2.470417000 1.218112000

H -5.340249000 -1.315123000 2.827235000

H -0.787059000 1.955609000 0.948744000

H 0.377635000 4.111949000 1.110348000

H 2.819223000 4.272516000 0.743116000

H 4.961947000 3.168680000 0.260658000

H 6.283817000 1.120095000 -0.127097000

H 2.727720000 -1.270269000 0.186302000

H -2.977576000 -1.982587000 -2.470814000

H -4.341582000 -2.908390000 -2.657200000

H 0.677568000 -0.971405000 -0.395838000

H 5.064542000 -1.901164000 -2.348984000

Cd -3.215576000 1.951709000 -1.051751000

**Cd^2+^**, ωB97XD/6-31+G(d,p)/Dyall-cvdz, in water

*G* = -5467.505494 hartree, HF = -5467.488799 hartree

Cd 0.000000000 0.000000000 0.000000000

**Pb^2+^**, ωB97XD/6-31+G(d,p)/Dyall-cvdz, in water

*G* = -19528.693699 hartree, HF = -19528.67615 hartree

Pb 0.000000000 0.000000000 0.000000000
